# TiO_2_-doped borate glass and glass-ceramic: properties and prospects for biological and electrical applications

**DOI:** 10.1038/s41598-025-03064-x

**Published:** 2025-06-03

**Authors:** Gehad Y. Abo El-Reesh, M. A. Azooz, M. A. Ouis, Amira A. Gamal, Reham M. M. Morsi, S. M. Abbas, S. Abd Elkhalik

**Affiliations:** 1 Chemistry Department, Faculty of Science, Beni-Suef University, Beni-Suef, Egypt; 2 Glass Research Department, National Research Centre, Dokki, Giza, Egypt; 3 Chemistry of Natural and Microbial Products Department, Pharmaceutical and Drug Industries Research Institute, National Research Centre, Dokki, Cairo, Egypt; 4 Physical Chemistry Department, National Research Centre, 33 El Bohoth St., Dokki, P.O. 12622, Giza, Egypt

**Keywords:** Borate glass, Glass-ceramic, TiO_2_, Antimicrobial, Electrical properties, Chemistry, Materials science

## Abstract

This study explores the synthesis, characterization, and potential applications of TiO_2_-doped borate glasses and their glass-ceramics, focusing on their biological and electrical properties. Examining the impact of varying the TiO_2_ content on the structural, electrical, and antimicrobial properties of the prepared samples was done. X-ray diffraction, Fourier-transform infrared spectroscopy (FTIR), density, and Field emission scanning electron microscope (FESEM) were employed to analyze the material’s structural integrity and phase transitions. The AC conductivity (σ_ac_) was measured within the frequency range of 0.042 kHz–1 MHz and at the temperature range of 298–573 (K). The estimated DC conductivity proved that incorporating of TiO_2_ at the expense of BaO results in higher conductivity values than those of the free glass and glass ceramic samples. The prepared samples exhibited a semiconducting nature. The dielectric constant (ɛʹ) values increase upon doping with TiO_2_. The incorporation of TiO_2_ improved the bioactivity (antimicrobial) of the studied glasses, making it suitable for biomedical applications such as drug delivery and tissue engineering. Also, the long-term stability and cytotoxicity were evaluated. The results indicate that TiO_2_-doped borate glasses and glass-ceramics present a promising avenue for the development of multifunctional materials that meet the demands of both biological and electrical applications.

## Introduction

Borate glass is considered as a wide glass former that has the ability to alter its properties depending on the type and quantity of the additives that may be incorporated into the glass composition^1,2^. B_2_O_3_ can exist as triangular [BO_3_] and tetrahedral [BO_4_] structural units in the glass matrix due to the ability of boron to alter its coordination number with oxygen^3,4^. Borate-based glass can be included in various applications such as radioactive waste immobilization, ionic conductors, smart windows manufacturing, plant nutrition agri-glass, high power solid state laser, and bioactive materials^5–11^. Glass can be used as a bioactive material through its use as bone fillers, toothpaste and composites, dental cement, treating burns and wounds, synthetic scaffolds and bioactive antibacterial materials^12–15^. Borate glass materials exhibit a higher level of bioactivity than silicates and this is attributed to their lower chemical durability, higher rate of hydroxy apatite (HA) conversion, and fast rate of dissolution in biological fluids^16,17^. They have also been exhibited to enhance wound healing, cell proliferation, and cell differentiation^18,19^. Borate glasses exhibit antibacterial effects and inhibition of several species of Gram-positive and Gram-negative bacteria^20,21^. Replacing SiO_2_ in the commercial glass system S53P4 with B_2_O_3_ with (25, 50, 75, and 100 mol%) shows improved antibacterial effects against E.coli bacteria compared to the base system^22^. Glass-ceramics can be obtained through the crystallization of glasses by thermal heat treatment. Bioactive glass-ceramics have been evaluated for their biological effects. As reported^23–25^, controlled crystallization of glass samples decreases their reaction kinetics with physiological solution ions without the inhibition of HA precipitation, even in the case of intensively crystallized glass-ceramics. Da silva et al. evaluated the effect of crystallization (as a function of heat treatment time) of the prepared glass ceramics on their bioactive and antibacterial properties^21^. The bacterial infections that arise from surgical procedure failure or nosocomial infections lead to bone implant failures during the treatment of bone fractures using glass. The activity of antibacterial glass can be enhanced through doping antibiotics^26^ or trace amounts of ions that can cause antibacterial effects that can be slowly released, e.g. silver (Ag^+^), copper (Cu^2+^), zinc (Zn^2+^), strontium (Sr^2+^) and titanium (Ti^2+^)^27–30^. Titanium is considered a 3d transition metal that can be added to the glass matrix to modify glass properties like electrical, thermal, structural, and optical properties^31–33^. In glass networks, titanium can present in both trivalent and tetra-valent oxidation states depending on the glass composition, the condition of melt, and its concentration^34,35^. In borate glass, titanium ions exist as a network former (TiO_4_ or B-O-Ti) and glass modifier (TiO_6_) groups^36^. The presence of a small amount of TiO_2_ in glass can improve glass’s chemical durability, forming the ability of glass to act as a nucleating agent during heat treatment^37–39^. Depending on the porosity of bioactive glass^40,41^ and the ability to adjust its reaction rate by altering its composition^42^, incorporated ions (TiO_2_) leach out of glass in a controlled delivery release over an extended range of time^43^. Titanium has been known to have its antibacterial effects and has also been included in different materials to inhibit the growth of bacteria^44–46^. Samudrala et al.^47^ produced a borosilicate glass composition doped with TiO_2_ to improve biological response in bone tissue engineering fields, and they observed antibacterial activity of 2% TiO_2_ in the system against S. aureus and E. coli. The electrical properties of glass-ceramics are significantly influenced by their composition and the controlled crystallization process. For instance, the material’s dielectric behavior in the BaO–Al_2_O_3_–SiO_2_–B_2_O_3_ system was affected by varying the amounts of B_2_O_3_, BaO, and crystallization temperatures^48^. Borate glasses doped with titanium dioxide (TiO_2_) in the system 20Na_2_O–(20 − x)CdO–60B_2_O_3_–xTiO_2_ (0 ≤ x ≤ 5 mol%), have garnered significant interest due to their distinctive electrical properties^49^. Further investigations into borate glasses within the B_2_O_3_–Na_2_O–TiO_2_ system have examined the effects of varying TiO_2_ concentrations on both optical and electrical properties. Dielectric measurements reveal that the dielectric constant (ε’) is influenced by the TiO_2_ content. Additionally, the direct current (DC) conductivity of these glasses is found to be temperature-dependent, with higher TiO_2_ concentrations leading to increased conductivity. These findings suggest that TiO_2_ acts as a modifier in the glass network, affecting the mobility of charge carriers and thus the electrical properties^50^. Enhancing the electrical and thermal expansion coefficient of borosilicate glasses doped with TiO_2_ and its counterpart glass ceramics was investigated^51^. Borate glass is generally insulating in nature; the addition of transition metal oxides (TMO), such as V_2_O_5_, Fe_2_O_3_, CuO, WO_3_, TiO_2_, and MoO_3_, to the glass, makes it an electronic conducting semiconductor^52^. The semiconducting properties of this type of glass are due to the ability of TMO to present more than one valence state^52^. Structural, electrical, thermal expansion coefficient (TEC), and hardness were studied to examine the impact of including transition metal ions on enhancing the electrical and TEC of borosilicate glasses doped with TiO_2_ and its counterpart glass ceramics. The prepared glass–ceramic was found to be suitable for use in electronics and solar cell applications based on its overall thermal and electrical properties^51^. The purposes of this study are: (1) Preparation of TiO_2_- modified glass samples by substitution of BaO in the glass system (40 B_2_O_3_-20 Al_2_O_3_-10 NaF-(30-X) BaO-X TiO_2_) mol % and their corresponding glass ceramics; (2) Characterization of the prepared samples by various tools including Fourier transform infrared spectroscopy (FT-IR), X-ray diffraction (XRD), Field emission scanning electron microscopy (FESEM), Density and Molar volume; (3) Studying the effect of gradual increasing of TiO_2_ mol% on structural, electrical and biological properties; (4) Electrical properties and dielectric behavior of these samples have been characterized, which may be suitable for use in electronic devices; (5) Evaluating the bioactivity of the materials to determine their suitability for biomedical applications, including antimicrobial activity.

## Experimental section

### Materials

All raw materials were used directly without previous purification. Orthoboric acid (H_3_BO_3_, Winlab, Leicestershire, UK, Assay 99.5%) for B_2_O_3_, anhydrous heavy barium carbonate (BaCO_3_, E. Merck, Darmstadt, Germany) for BaO, Titanium dioxide (Fluka, Germany) for TiO_2_, Aluminium oxide (Fluka, chemei AG, switzer land) and Sodium fluoride.

### Synthesis of materials

#### Glass synthesis

In this study, a traditional melt annealing method was employed for the preparation of the glass system (B_2_O_3_-Al_2_O_3_-NaF-BaO-TiO_2_) where a series of four TiO_2_- modified glass compositions were prepared by replacing BaO in the glass composition with TiO_2_. The glass batch was weighed accurately and put into a platinum crucible. The melt was synthesized in an electric furnace at 1375 °C for 2 h. The melts were continuously rotated at time intervals to maintain the homogeneity of melts and get rid of bubbles. Stainless steel molds were pre-heated to cast the melt and then transferred at once into a muffle furnace optimized at 400 °C for annealing for 1 h, then left to cool to room temperature. The symbol of the base glass is BG0, whereas the symbols of the four modified glasses are BG1, BG2, BG3, and BG4, respectively. Table 1 represents the glass composition in mol%.

**Table 1:** Composition of base glass and the series of TiO_2_ modified glass in mol%.

Glass code	B_2_O_3_	Al_2_O_3_	NaF	BaO	TiO_2_
BG0	40	20	10	30	–
BG1	40	20	10	29.5	0.5
BG2	40	20	10	29	1
BG3	40	20	10	28.5	1.5
BG4	40	20	10	28	2

#### Glass-ceramic synthesis

The glass-ceramic materials were synthesized through thermal heat treatment of the parent prepared glass samples. First, the glass samples were heated to the temperature of nucleation (480 °C) at a rate of 5 °C/min for 4 h. Then, the temperature was elevated to the crystal growth temperature (750 °C) at a rate of 5 °C/min, and the samples were kept at this temperature for 6 h. The samples were left to cool at a rate of 25 °C/min to room temperature. The symbol of the base glass-ceramic is BGC0 where, the symbols of the four modified glass-ceramics are BGC1, BGC2, BGC3, and BGC4, respectively.

### Xray diffraction analysis

X-ray diffraction (XRD) patterns were obtained for glass-ceramic samples with a PANalytical (Empyrean) X-ray diffraction using Cu K α radiation (wave length 0.154 cm^− 1^) at an accelerating voltage of 40 KV, current of 35 mA, scan angle range from 20° to 70° and scan step 0:02°.

### FTIR spectral measurements

The Fourier transform infrared (FT-IR) spectra (4000 –400 cm^− 1^) were obtained by a Bruker (Vertex 70) spectrometer for both glass and glass-ceramic samples using the KBr technique. The powder samples were mixed with pulverized KBr at a ratio of 1:100 mg. The mixtures were pressured at 5 tons/cm^2^ to produce homogenized discs. Immediately, the prepared discs were measured.

### Density measurements

Density was done through the standard Archimedes method at room temperature, with distilled water as an immersion liquid. Three samples of each glass were employed to calculate the density. The values are precise to ± 0.02 g/cm^3^. Density can be calculated by using the Eq. (1)

1 $${\uprho } = {\text{W}}_{{\text{a}}} {\uprho }_{{\text{w}}} /\left( {{\text{W}}_{{\text{a}}} - {\text{W}}_{{\text{w}}} } \right)$$

Where ρ is the density of the glass sample, W_a_ and W_w_ are the weights of the glass sample in both air and distilled water, respectively, and ρ_w_ is the density of distilled water.

The molar volume, which is known as the volume of one-gram mole of glass, can be calculated by the relation (2).

2 $${\text{V}}_{{\text{m}}} = 1/{\uprho }\sum {\text{X}}_{{\text{i}}} {\text{M}}_{{\text{i}}}$$

Where, X_i_ M_i_ are the components of molar fraction and molecular weight.

### **Field emission scanning Electron microscope (FESEM) images**

The morphology of the glass-ceramic samples was examined by SEM type (JEOL- JSM- T2O Japan) after coating them with surface layers of gold.

### Electrical measurements

The AC conductivity (σ_ac_) of the prepared samples was measured using the LRC Hi-Tester (HIOKI, 3532–50), Japan, over a frequency range from 0.042 kHz to 1 MHz and a temperature range from 298 to 573 (K). Increasing the temperature was provided by increasing the input voltage of a variac transformer connected to a wire-wound resistance heater. The temperature was determined using a copper/Constantine thermocouple in proximity to the sample. The AC conductivity, σ_ac_, is calculated using the relation (3).

3 $${\upsigma }_{{{\text{ac}}}} = {\upomega \upvarepsilon }_{{\text{o}}} {\upvarepsilon }^{\prime } \tan{\updelta }$$

Where ω is the angular frequency, ε_o_ is the permittivity of free space, equals 8.85 × 10^− 12^ F m ^− 1^, and ε′ is the dielectric constant which is determined from expression (4).

4 $${\upvarepsilon }^{\prime } = {\text{Cd}}/{\upvarepsilon }_{{\text{o}}} {\text{A}}$$

Where d represents the thickness of the sample, and A represents the sample surface area. The capacitance, C, and the dissipation factor, tan δ, are obtained directly from the instrument for the studied samples^53^.

### Biological activities experiments

#### Microbial strains

Five microorganisms were selected for testing the antimicrobial activity. Two G + ve strains (*Staphylococcus aureus*, *Bacillus cereus*), one G-ve bacteria strain *(Escherichia coli)*, one yeast (*Candida albicans*), and one fungus (*Aspergillus niger)* were obtained as clinical strains from El-Demerdash hospital, Cairo, Egypt.

#### Inoculum preparation

All used media were sterilized for inoculum preparation; nutrient broth medium was used for bacteria while potato dextrose broth medium was used for fungus and yeast. The tubes were incubated for 24 h at 37 °C for bacteria and 2–3 days at 30 °C for yeast and fungi.

#### Determination of antimicrobial assay

The ten samples (glass) were in vitro evaluated for their antimicrobial activity. Standard and clinically isolated microorganism strains were used for antimicrobial assays such as *E. coli* (Gram-negative bacteria), *Bacillus cereus* (Gram-positive bacteria), *Staphylococcus aureus* (Gram-positive bacteria), *Candida albicans* (yeast), and *Aspergillus niger* (fungi) by measuring the growth inhibition of pathogenic organisms according to^54^. Samples (0.03 g) were added to the tubes [10 mL of nutrient broth (bacteria and yeast) or potato dextrose broth (fungi)]. The inoculated tubes (100 µl from the inoculum) and standard tubes (without adding samples) were incubated for 24 h at their optimum growth temperatures (37 °C for bacteria and 28 °C for yeast and fungi), and the optical densities (O.D) of the microbial growth were measured by spectrophotometer at 600 nm. Experiments were carried out in triplicate for each strain of the evaluated microorganisms. The reported results are the average.

$${\text{N}}{\text{.B: Inhibition}}\,{\text{of}}\,{\text{microbial}}\,{\text{growth}}\,{\text{(\% ) = 100}} - {\text{[(O}}{\text{.D}}\,{\text{of}}\,{\text{sample}}\,{\text{(trial)/O}}{\text{.D}}\,{\text{of}}\,{\text{standard) }}$$

#### Longer-term stability assessments

To evaluate the long-term antimicrobial stability of the glass and glass ceramic samples, the same antimicrobial assay protocol described previously was applied. However, in this case, the samples had been stored under ambient laboratory conditions for approximately one year before testing. The test was carried out against the same microbial strains using identical incubation conditions.

#### Cytotoxicity assay

**Mammalian cell lines: HFB 4** cells (Human normal melanocytes cell line) were obtained from the American Type Culture Collection (ATCC, Rockville, MD). **Chemicals Used**: Dimethyl sulfoxide (DMSO), Fetal Bovine serum, MTT and trypan blue dye were purchased from Sigma (St. Louis, Mo., USA). DMEM, HEPES buffer solution, L-glutamine, gentamycin and 0.25% Trypsin-EDTA were purchased from Lonza (Belgium).

**Cell line propagation** The cells were propagated in Dulbecco’s modified Eagle’s medium (DMEM) supplemented with 10% heat-inactivated fetal bovine serum, 1% L-glutamine, HEPES buffer, and 50 µg/ml gentamycin. All cells were maintained at 37ºC in a humidified atmosphere with 5% CO_2_ and subcultured two times a week.

**Cytotoxicity evaluation using viability assay** For cytotoxicity assays, the cells were suspended in medium at concentration 1 × 10^5^ cell/well in Corning^®^ 96-well tissue culture plates, and incubated for 24 h. The tested compounds were then added into 96-well plates (three replicates) to achieve eight concentrations for each compound. Six vehicle controls with media or PBS were run for each 96-well plate as a control. After incubating for 24 h, the numbers of viable cells were determined by the MTT test. Briefly, the media was removed from the 96-well plate and replaced with 100 µl of fresh culture DMEM medium without phenol red, then 10 µl of the 12 mM MTT stock solution (5 mg of MTT in 1 mL of PBS) to each well, including the untreated controls. The 96-well plates were then incubated at 37 °C and 5% CO_2_ for 4 h. An 85 µl aliquot of the media was removed from the wells, and 50 µl of DMSO was added to each well and mixed thoroughly with the pipette and incubated at 37 °C for 10 min. Then, the optical density was measured at 590 nm with the microplate reader (SunRise, TECAN, Inc, USA) to determine the number of viable cells, and the percentage of viability was calculated through Eq. 5.

5 $${\text{[(ODt/ODc)]}} \times {{100\% }}$$

Where ODt is the mean optical density of wells treated with the tested sample and ODc is the mean optical density of untreated cells. The Cytotoxic concentration (CC_50_), the concentration required to cause toxic effects in 50% of intact cells, was estimated from graphic plots of the dose response curve for each conc. Using Graphpad Prism software (San Diego, CA. USA).

## Results and discussion

### X-ray diffraction measurements

**Fig. 1:**
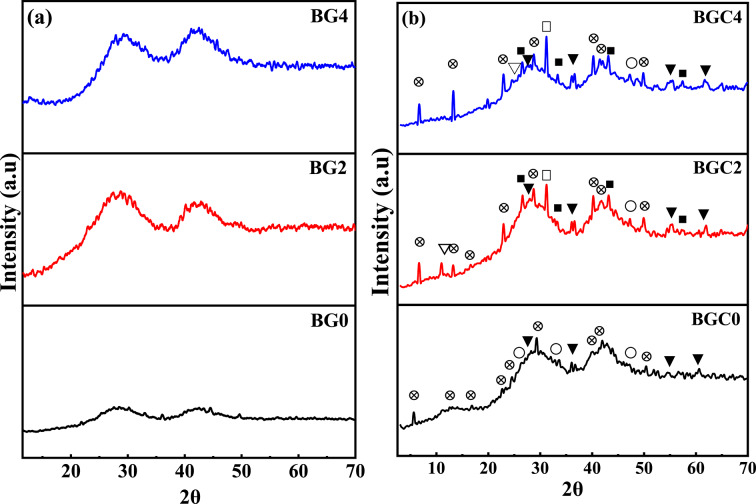
XRD patterns of three selected (**a**) glass samples (BG0, BG2 and BG4) and (**b**) their corresponding glass ceramics (BGC0, BGC2 and BGC4 where, ⊗ Ba_3_B_2_O_6_, ○ Ba_5_B_4_O_11_, ▼AlBaBO_3_F_2_, ▽Al_2_O_3_, ■ BaTi_2_O_5,_ □ BaTiO_3_.

Figure 1 Exhibits XRD patterns of three selected glass samples (BG0, BG2, and BG4) and their corresponding crystallized samples (glass-ceramics) (BGC0, BGC2, and BGC4). Figure 1a displayed broad humps arising from the amorphous nature of the prepared glass samples. As shown in Fig. 1b, certain crystalline phases were separated through heat treatment of the parent glass samples. These crystalline phases are Ba_3_B_2_O_6_ (card no 96-156-8109), Ba_5_B_4_O_11_, AlBaBO_3_F_2_ (card no 96-152-6215), and Al_2_O_3_ in addition to BaTi_2_O_5_ and BaTiO_3_ phases related to incorporation of TiO_2_ into the matrix (in BGC2 and BGC4 samples). For BGC0, broad humps with weak intensity peaks of crystalline phases were observed. Since TiO_2_ acts as a nucleating agent^55^, the gradual increase of TiO_2_ content caused an increase in peaks intensity of the formed crystalline phases, indicating continuous growth of crystal grains in the glass matrix^56^. The quantitative crystalline phases composition is displayed in Table 2.

**Table 2:** The quantitative crystalline phases composition.

Samples code	Crystalline phases %					
	Ba_3_B_2_O_**6**_	Ba_5_**B**_**4**_**O**_**11**_	AlBaBO_3_**F**_**2**_	A_2_lO_3_	BaT_2_iO_5_	BaTiO_3_
BGC0	54	24	32	-	-	-
BGC2	40	5	17	7	16	15
BGC4	43	6	19	-	17	15

### FTIR study

**Fig. 2:**
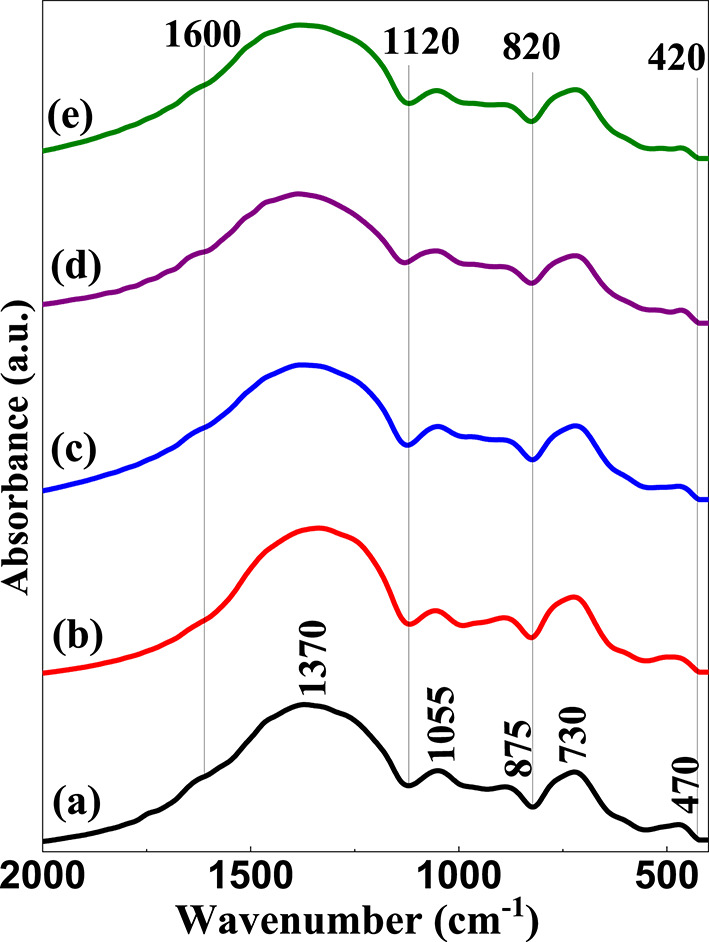
FTIR absorption spectra of base and doped TiO_2_ borate glass samples where, (**a**) BG0, (**b**) BG1, (**c**) BG2, (**d**) BG3, (**e**) BG4.

Figure 2 shows the FTIR spectra over a range from 2000 to 400 cm^− 1^ of the base and doped TiO_2_ borate glass samples. The figure exhibits the characteristic vibrational bands of both triangular and tetrahedral borate groups in their specific vibrational regions. As shown in Fig. 2, the mid infrared region (1600–400 cm^− 1^) is the region where B-O vibrations are dominant. This region can be distinguished into three broad bands^57–61^, which are:

(I) The strong vibrational band ranging from (1120–1600 cm^− 1^) appeared due to the stretching vibrations of triangular borate units (BO_3_ and BO_2_O^−^).
(II) The absorption band from (820–1120 cm^− 1^) is assigned to the symmetric and asymmetric stretching of B-O in BO_4_ tetrahedral group units.
(III) The area ranging from (420–820 cm^− 1^) is related to the bending vibrations of B-O-B in [BO_3_ and BO_4_] units, B-O-Ti and TiO_4_^62^.

**Fig. 3:**
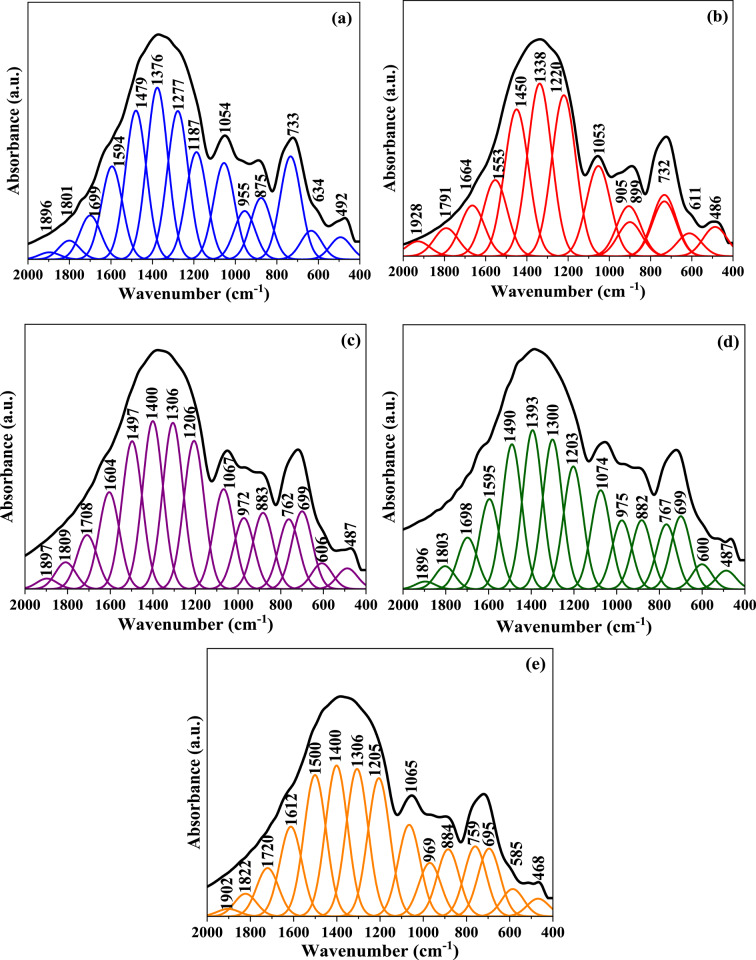
Deconvoluted IR spectra of (**a**) BG0, (**b**) BG1, (**c**) BG2, (**d**) BG3, (**e**) BG4.

Figure 3 shows the FTIR spectra of all synthesized glass samples and their spectra deconvolution. Deconvolution of the bands gives a good chance for a more precise explanation of the overlapping bands. As expected, the FTIR spectra of these glass samples have both triangular BO_3_ and tetrahedral BO_4_ vibrational bands and the absorption band intensities caused by tetrahedral borate groups [BO_4_] at (820–1120 cm^− 1^) are lower than that caused by triangular borate groups [BO_3_] at (1120–1600 cm^− 1^). This is attributed to the presence of alkali (NaF) and alkaline earth oxides (BaO) that can transform BO_3_ group units into BO_4_ units to a certain limit (16–20 mol%), after which the surplus of BaO supports the formation of non-bridging oxygen^63^. These results were also obtained by Kamitsos in his study on alkali oxides (LiO_2_, K_2_O, Na_2_O) and alkaline earth oxides (BaO, SrO, CaO) in borate glass^64^. The FTIR spectral curves of doped TiO_2_ borate glass samples (BG1, BG2, BG3, and BG4) are noticed to be closely similar to the corresponding base sample (BG0) with the same characteristic broad bands but with some shifts to lower or higher wavenumber of some peaks caused by the addition of TiO_2_ into glass composition^35,65^. As explained by Marzouk et al.^62^, the vibrational bands of crystalline TiO_2_ are located within the wavenumber range from 400 to 800 cm^− 1^, which is the same region of appearance of borate groups bending vibrations, indicating that vibrations of TiO_2_ doped glass at (400–800 cm^− 1^) are composite of merged bands which contain overlapping BO_3_, BO_4_, B-O-Ti, and TiO_4_ vibrations. It is worth mentioning that introducing TiO_2_ into glass composition creates some observed changes which can be discussed as follows^1,62,66,67^:

(I) The band at about 492 cm^− 1^, which is related to O-B-O and vibrations of metal cations (Ba^2+^) shifts to a lower wavenumber at about (468–487 cm^− 1^) and this is attributed to vibrations of added Ti^2+^.
(II) The vibrational band located at 634 cm^− 1^, which is identified due to the in-plane bending of BO_3_ units, shifts to a lower wavenumber at about (585–611 cm^− 1^), in addition to the appearance of a new band in (BG2, BG3, and BG4) at (695–699 cm^− 1^) on the gradual increasing of TiO_2_ content. This band can be assigned to asymmetric (Ti-O-Ti) stretching vibrations of TiO_6_.
(III) The vibrational absorption peak at 733 cm^− 1^, which refers to the bending vibration of the B-O-B linkage, is completely overlapped with the band of TiO_4_ (B-O-Ti) bending, leading to the creation of the overlapped band at (759–767 cm^− 1^).

**Fig. 4:**
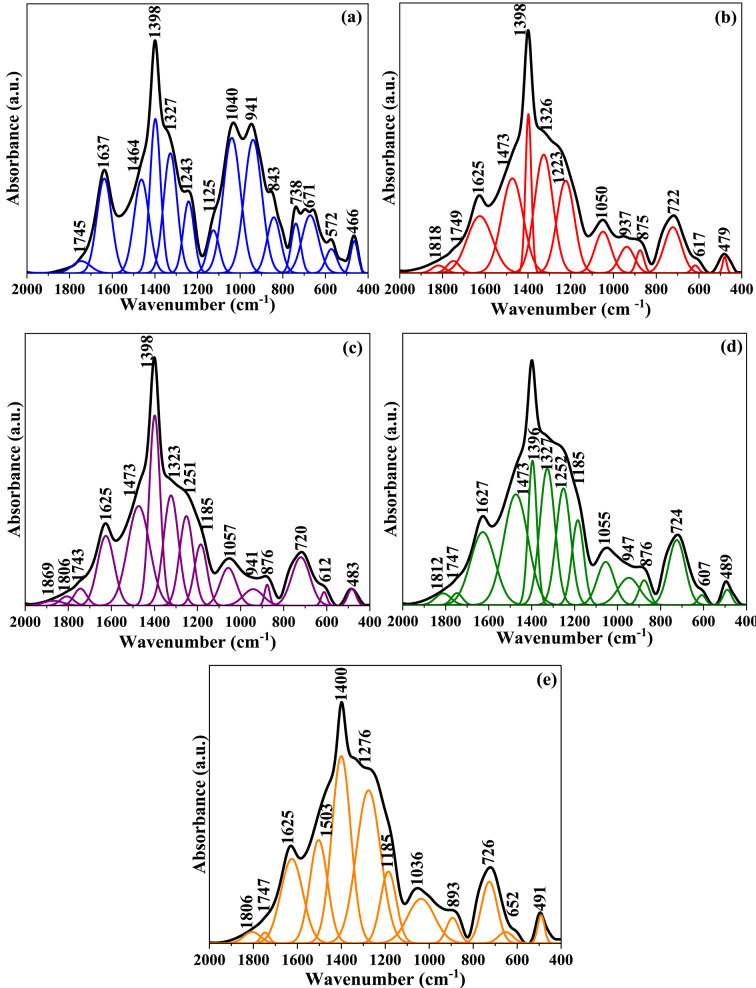
Deconvoluted IR spectra of (**a**) BGC0, (**b**) BGC1, (**c**) BGC2, (**d**) BGC3, (**e**) BGC4.

From the above assumptions, one can conclude that at lower concentrations of TiO_2_, TiO_4_ (network former) structural units are mainly present in the network and there is a gradual increase in the formation of TiO_6_ (network modifier) structural units with an increase in TiO_2_ content. The assignments of the observed vibrational absorption bands are summarized in Table 3. Figure 4 shows FTIR spectra of glass-ceramic samples. The FTIR displays the same characteristic spectral bands as their parent glasses, but these vibration modes are noticed to be sharper with split peaks related to the fine crystalline derivatives of three and four co-ordinated borate groups^68^.

**Table 3:** Assignments of the observed vibrational absorption bands^69–74^.

Peak no.	Wavenumber (cm^− 1^)					Assignments
	BG0	BG1	BG2	BG3	BG4	
1	492	486	487	487	487	Vibrations of metal cations Ba^2+^, Ti^2+^
2	634	611	606	600	585	In plane bending vibrations of BO_3_ units.
3	-	-	699	699	695	asymmetric (Ti-O-Ti) stretching vibrations of TiO_6_
4	733	732	762	767	759	bending vibration of B-O-B linkage and TiO_4_ (B-O-Ti) bending
5	875	899	883	882	884	BO_4_ stretching of tri-, penta, and diborate groups
6	955	905	972	975	969	
7	1054	1053	1067	1074	1065	
8	1187	1220	1206	1203	1205	Asymmetric stretching vibrations of (BO_3_, BO_2_O−)
9	1277	-	1306	1300	1306	
10	1376	1338	1400	1393	1400	
11	1479	1450	1497	1490	1500	
12	1594	1553	1604	1595	1612	
13	1699	1664	1708	1698	1720	Vibrations of water, OH groups.
14	1801	1791	1809	1803	1822	
15	1896	1928	1897	1896	1902	

### Density measurements

**Fig. 5:**
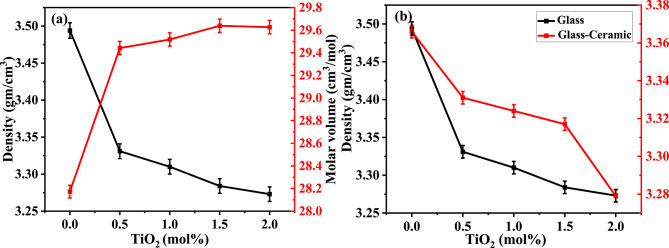
Density and molar volume of glass (**a**) and density of glass and glass-ceramics (**b**).

Density is considered the simplest tool that can help to understand the changes occurring in the structure of the glass network. The density depends on the compactness, the geometrical configurations, the coordination number, the cross-link densities, and the dimensions of the interstitial spaces of the glass^75^. The influence of the substitution of TiO_2_ at the expense of BaO on the measured density and the calculated molar volume of the studied glass system is demonstrated in Fig. 5 a. As illustrated, the substitution of BaO by TiO_2_ caused a slight decrease in density from 3.494 g/cm^3^ to 3.273 g/cm^3^, this can be mainly attributed to the lower molecular weight of TiO_2_ (79.867 g/mol) than BaO (153.33 g/mol). Glass density is directly proportional to the molecular weight of glass and inversely proportional to molar volume (related to ionic radius). Since the ionic radius of Ti^2+^ is smaller than Ba^2+^, the overall decrease in density indicates that the molecular weight change is more effective on density than the ionic radius change^76^. The molar volume of glass linearly increased from 28.173 cm^3^/mol to 29.627 cm^3^/mol with increasing TiO_2_ in the glass matrix as shown in Fig. 5a. As expected, the density and molar volume of glass exhibited opposite behaviour. The volume expansion may be related to the gradual formation of octahedral TiO_6_ structural units by increasing TiO_2_ content in the glass matrix (as deduced from the FTIR study). These TiO_6_ units act as a network modifier and hence increase the amount of non-bridging oxygen which produces an open structure and increase the interstitial positions in the glass network^37,38,77^. Also, the molar volume increase may be associated with the increase in the total number of oxygen atoms by substitution of BaO by TiO_2_, leading to the expansion of the glass network^76^. As shown in Fig. 5b, the density of the synthesized glass samples is lower than that of their corresponding glass-ceramics. According to^78,79^, heat treatment relaxes the glass structure by releasing some of its internal energy, the thing which causes rearrangement and compactness of the structure. All the values of density, molar mass, and molar volume of glass, and the density of glass ceramics are listed in Table 4.

**Table 4:** Density and molar volume of glass and glass-ceramics.

Samples code		Density (g/cm^3^)	Molar mass (g/mol)	Molar Volume (cm^3^/mol)	Samples code		Density (g/cm^3^)
Glass	BG0	3.494	98.438	28.173	Glass-Ceramics	BGC0	3.366
	BG1	3.331	98.0707	29.442		BGC1	3.331
	BG2	3.310	97.7034	29.518		BGC2	3.324
	BG3	3.284	97.3361	29.639		BGC3	3.317
	BG4	3.273	96.9688	29.627		BGC4	3.279

### Scanning electron microscope (SEM) images

**Fig. 6:**
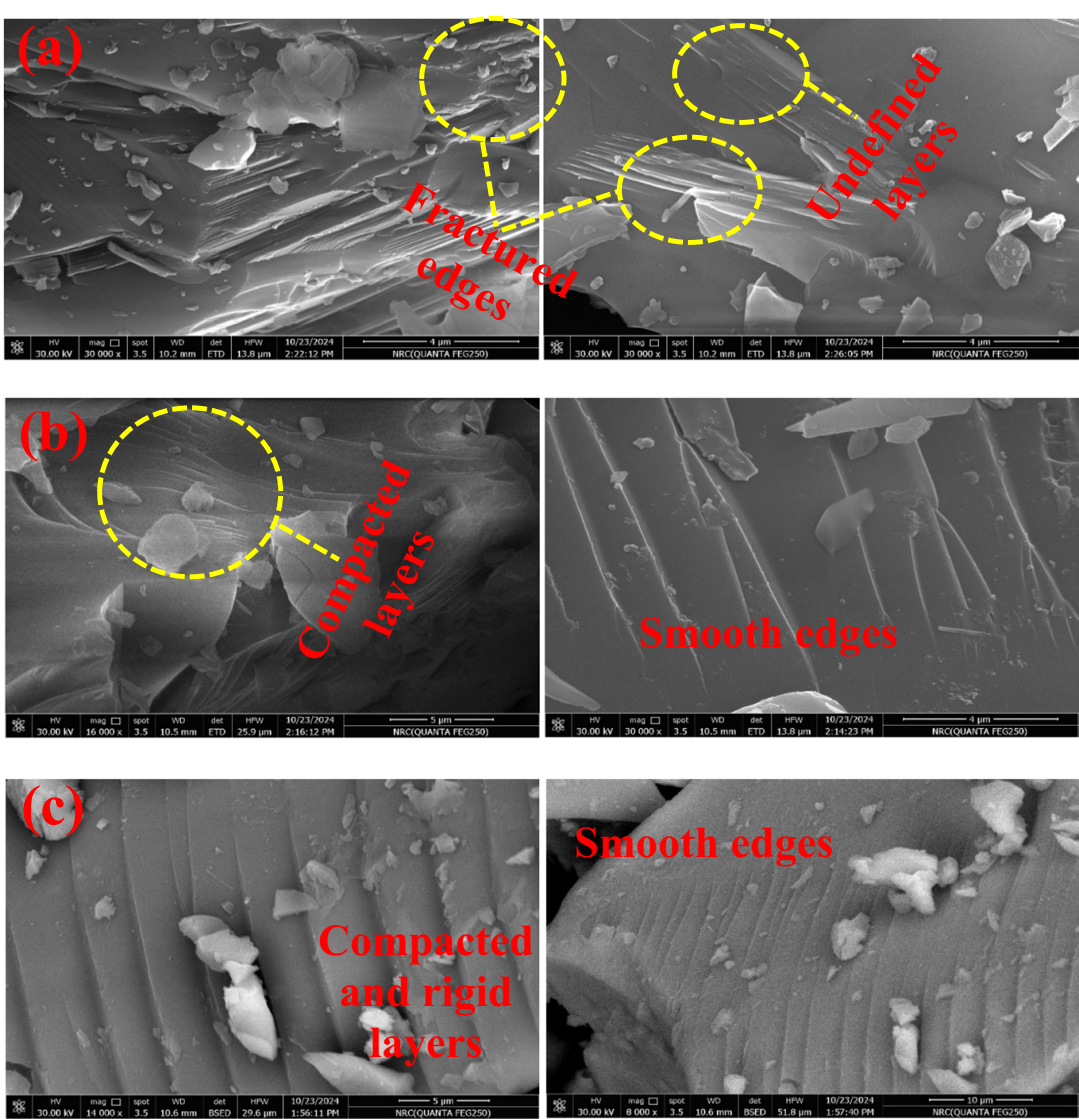
SEM micrographs of three selected (**a**) BGC0, (**b**) BGC2 and (**c**) BGC4.

Figure 6 shows the SEM micrographs of three selected BGC0, BGC2 and BGC4 glass-ceramic samples. It is obvious that the micrographs of all the selected samples exhibit a crystalline structure (as supported by XRD data) which consists of multiple layers (sheets). Unlike the base one BGC0 (without TiO_2_), increasing TiO_2_ content makes the layers more compacted, defined and rigid with smooth edges (layer edges without fractures).

### Electrical measurements

**Fig. 7:**
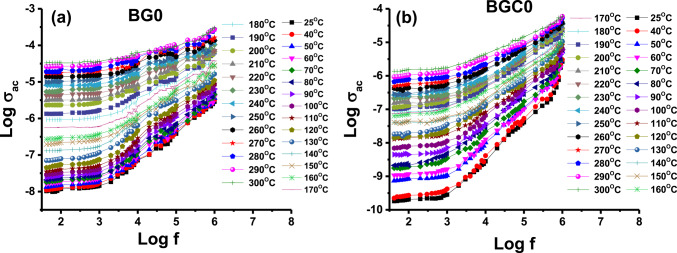
Variation of log σ_ac_ as a function of applied frequency for samples (**a**) BG0 and (**b**) BGC0 at different temperatures.

Figure 7 illustrates the frequency dependence of the alternating-current conductivity (σ_ac_) at different temperatures in the frequency range extending from 0.042 kHz to 1 MHz for samples BG0 and BGC0 (as representative samples). It can be observed that the conductivity σ_ac_ increases with increasing frequency. This observation suggests that the electrical conductivity of the material is a thermally activated process^80^. The increase in AC conductivity of the material with increasing frequency can be attributed to the release of space charges caused by a decrease in the material’s barrier properties^81^. The variation observed in Fig. 7 reveals that the conductivity increases strongly with frequency at lower temperatures, while the increase is lower at higher temperatures. It is generally known^82^ that AC conductivity is influenced by both the polarization of immobile ions (e.g., Al^2+^, B^2+^) within the borate network and the mobility of charge carriers. The conductivity due to charge carriers is small at low temperatures. Accordingly, the AC conductivity is expected to exhibit the observed frequency dependence (Fig. 7), science depends mainly on the polarization of immobile ions. The conductivity of the charge carriers is expected to increase with temperature^83^. At high temperatures, the dominant factor in the conductivity will be the mobility of the charge carriers, while the polarization of the immobile ions will make a less significant contribution. Therefore, at high temperatures, the AC conductivity will have a lower dependence on frequency as shown in (Fig. 7). For example, the σ_ac_ of the sample BGC0 at 25 °C increases from 2.84 × 10^− 10^ to 2.65 × 10^− 6^ (S.cm^− 1^) at 1 kHz and 1 MHz, respectively, and at 300 °C increases from 1.73 × 10^− 6^ to 6.03 × 10^− 5^ (S.cm^− 1^) at 1 kHz and 1 MHz, respectively. The DC part is independent of frequency, is dominant at low frequencies and appears as a flat DC plateau at low-frequency regions. By extrapolating the plateau regions observed in Fig. 7, to zero frequency, the DC conductivity values of the samples can be estimated^84^. The DC electrical conductivity (σ_dc_) was plotted against the reciprocal temperature for all samples studied as shown in Fig. 9.

**Fig. 8:**
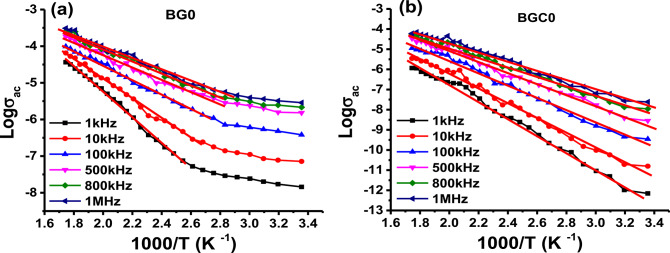
Variation of AC conductivity (σ_ac_) with reciprocal of temperature for samples (**a**) BG0 and (**b**) BGC0 at different frequencies.

Figure 8a, b shows the variations in AC conductivity as a function of the reciprocal of the temperature at different frequencies for un-doped glass and glass ceramic samples (as representative samples). It is noticed that the conductivity increases with increasing temperature, which indicates that they have semiconducting properties^85^. The values of the activation energies associated with AC conduction (E_a(ac)_) were calculated, in the high-temperature region, from the slopes of the linear fit (Fig. 8) and listed in Table 5. These values are in the range of 0.317–0.693 (eV) and 0.172–0.446 (eV) for glass and glass ceramic samples, respectively. It is also observed from Fig. 8, and the data in Table 5, that the slopes of the curves decrease as frequency rises. Consequently, the E_a(ac)_ values decrease as the applied field frequency increases, which may be attributable to electron jump-enhancement between localized states^86^.

**Fig. 9:**
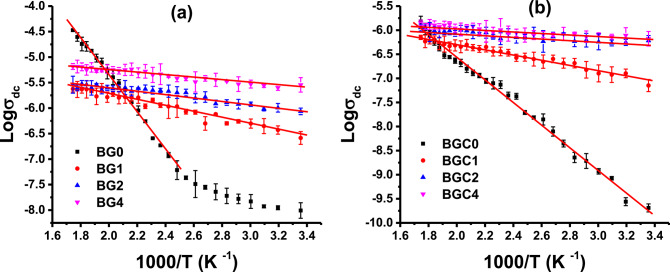
Variation of DC conductivity (σ_dc_) as a function of the reciprocal temperature for all samples (**a**) glasses and (**b**) glass ceramics at room temperature.

Figure 9 shows the temperature dependence of DC conductivity (σ_dc_) of all glass samples with the composition 40B_2_O_3_-20Al_2_O_3_-10 NaF-(30-x)BaO- xTiO_2_ and their corresponding glass ceramics, featuring varying TiO_2_ content in the temperature range 25°–300 °C. It is found that the estimated σ_dc_ increases with increasing temperature across the entire temperature range, indicating their semiconducting nature^87^. The investigated samples show a linear dependence of the logarithm of conductivity (log σ_dc_) on the reciprocal of absolute temperature (1/T), Fig. 9. This feature reveals the thermally activated mobility of the charge carriers according to the hopping conduction mechanism^88^ that can be described by the Arrhenius relation (6).

6 $${\upsigma }_{{{\text{dc}}}} = {\upsigma }_{0} \exp \left( { - {\text{E}}_{{\text{a}}} /{\text{kT}}} \right)$$

Where σ_0_ is the pre-exponential factor, E_a_ is the activation energy for the conduction process, k is the Boltzmann’s constant and T is the absolute temperature. In the high-temperature region ranging from 130 to 300 (°C), the DC activation energies (E_a(dc)_) are determined from the slopes of the straight lines of the log σ_dc_ plots versus 1000/T plots and the values are listed in Table 6. The range of activation energy values falls in the range of 0.053– 0.728 (eV) and 0.037 − 0.462 (eV) for glass and glass ceramic samples, respectively. From Fig. 9a, it can be noticed that the σ_dc_ of the sample BG0 exhibits a non-linear increase with the measuring temperature rising, featuring two distinct regions. These two regions are formed by two different mechanisms. The first region, at lower temperatures ranging from room temperature up to approximately 110 °C, is characterized by low activation energy (0.156 eV). This behavior was attributed to electronic conduction^89^, which may occur via small polaron hopping between defect states, such as oxygen vacancies that can present in the structure of amorphous glass systems^90^. While the second mechanism observed at higher temperatures (130–300 °C) exhibits higher activation energy. This is primarily due to the predominance of ionic movement mechanisms driven by the mobility of charge carriers, specifically Na^+^ ions.

Furthermore, for doped glass samples and for all glass ceramics, it is evident that the electrical conductivity (σ_dc_) increases linearly with rising temperature, accompanied by relatively lower activation energies than the parent glass (Table 6). This behaviour can be attributed to the electronic mechanism coming from the electronic transition between Ti^3+^ and Ti^4+^, which may exist by introducing TiO_2_ to the samples and increasing its content. These ions may participate in the increase of conductivity and decrease the activation energy values (Table 6). The transfer of these electrons could be considered as a small polaron hopping process^91^. In the glass ceramic samples (Fig. 9b), in addition to the above explanation, the participation of electronic conduction that lowers the E_a(dc)_ values may also come from the fact that within the bulk sample, the oxygen vacancies are usually created during heat treatment due to the loss of oxygen which are usually created during sintering and brings free electrons behind, making an n-type material^92^. When the temperature is increased, the created electrons become conduction electrons due to thermal activation energy^93^. The decrease of the E_a(dc)_ by doping with TiO_2_ suggests that the conduction in doped samples is mainly electronic.

**Fig. 10:**
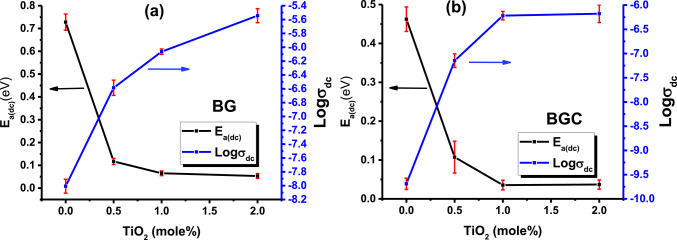
The variation of the σ_dc_ at 298 K, and the activation energy (E_a(dc)_) as a function of mole fractions of TiO_2_ for all the investigated samples (**a**) glasses (**b**) glass ceramics.

Variations of both room temperature DC electrical conductivity (σ_dc_) and activation energies (E_a(dc)_) as a function of TiO_2_ concentration (in mol%) are represented in Fig. 10. It is evident from this figure that the σ_dc_ increases with increasing TiO_2_ content in glass as well as glass ceramics. This increase may be attributed to an increase in the number of non-bridging oxygen (NBO) and, as a result, increasing in the open structure through which the charge carriers can move with higher mobility^94,95^. These results are in good agreement with the FTIR results, where increasing TiO_2_ content at the expense of BaO, supports the formation of non-bridging oxygen. Virender Kundu et al. reported that the formation of NBOs causes a decrease in band gab energy and, as a result, the DC conductivity increases^96^. Therefore, such structural changes can have an important influence on the mobility of Na^+^, which has the same concentration in all the samples under investigation, wherein the Na^+^ mobility increases in the presence of non-bridging oxygen atoms^97,98^. Besides, the electron hopping between Ti^4+^ and Ti^3+^ ions as referred to before. These conditions may contribute to an increase in conductivity with the incorporation of TiO_2_. For example, σ_dc_ at room temperature increases from 9.82 × 10^− 9^ to 2.86 × 10^− 6^ (S.cm^− 1^) and from 2.04 × 10^− 10^ to 6.64 × 10^−^7 (S.cm^− 1^) for glasses and glass ceramic, respectively, as the TiO_2_ concentration increases from 0 to 2 mol%. Notably (Fig. 10b), in the case of glass ceramic samples, the increase in conductivity seems to be nearly constant at higher contents of TiO_2_ (x = 1.5, 2 mol %). This behavior can be due to the compactness and rigidity of the glass ceramic structure produced by increasing TiO_2_ content that confirmed by SEM. This may affect the mobility of the charge carriers and, consequently, the rate of increasing conductivity is retarded. Also, in doped samples, the values of activation energy (E_a(dc)_) (Table 6) reveal the dominance of the electronic mechanism over the ionic transfer one.

Additionally, it is obvious (Table 6) that the σ_dc_ of the parent glass sample BG0 is higher than that of its glass ceramic BGC0. This can also be attributed to the rigid and dense structure caused by glass crystallization, which further hinders charge transport and decreases the conductivity values of glass ceramic than that of glass. This behavior follows that reported in other literature^99^. Furthermore, it was reported that semiconductors have conductivity values between 10^3^ and 10^− 8^ S.cm^− 1^^100^. Since the studied samples doped with TiO_2_ have room temperature conductivity values in the range 10^− 8^ – 10^− 6^ S/cm and exhibited a semiconducting nature; therefore, the doped samples studied can be considered as semiconducting materials.

**Fig. 11:**
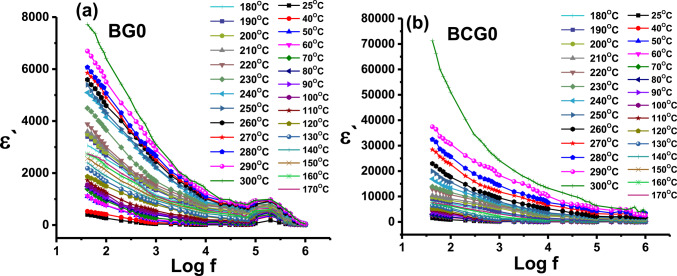
Variation of dielectric constant (ε′) versus frequency for samples (**a**) BG0 and (**b**) BGC0 at different temperatures.

The dielectric constant values are plotted as a function of frequency for samples BG0 and BGC0 (as representative samples) at different temperatures in Fig. 11. It can be seen that the samples showed high values of the dielectric constant at low frequency and high temperature. The higher ɛʹ values at lower frequencies were attributed to the contribution of the multi-component polarizability (i.e. electronic, ionic, orientation, and space charge)^101^. Interfacial contains defects such as vacancies and vacancy clusters. Thus, the space charges can move under the application of an external field and when they are trapped by the defects, lots of dipoles (space charge polarization) are formed^102,103^. Whereas, as the frequency increases, the dipoles are no longer able to rotate and their oscillation begins to lag behind the field, so the dielectric constant decreases and approaches nearly a constant value at high frequency^104^.

The increase in ε ′ with temperature is predicted to occur due to the weakening of the binding force between molecules/atoms with the increase in temperature, permitting the molecules/ atoms to vibrate more and more, which in turn increases the polarization, hence an increase in the dielectric constant ε ′^105,106^.

**Fig. 12:**
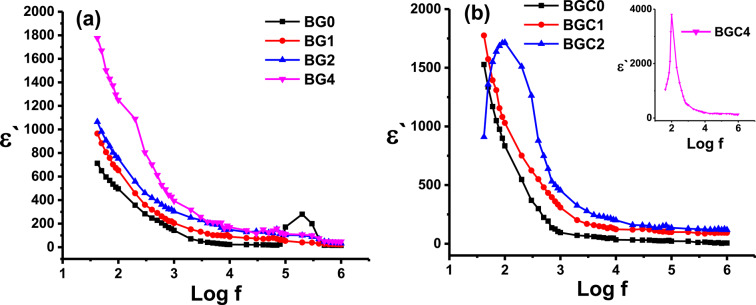
Variation of dielectric constant (ɛʹ) as a function of frequency for all samples (**a**) glasses and (**b**) glass ceramics at room temperature.

Figure 12 shows the frequency dependence of the dielectric constant (ɛʹ) measured at room temperature (298 K) for all samples studied. It can be observed that the studied samples exhibit high dielectric constants at low frequencies, which decreases with increasing frequency. Similar behavior was reported in some amorphous materials^107^. Moreover, it can be noticed that the ɛʹ is increased by doping TiO_2_ and increasing its content. This behaviour can be explained based on the structural changes which produced the gradual formation of octahedral TiO_6_ structural units by the incorporation of TiO_2_ that cause the creation of defective dipoles within the glass network^108^ in addition to introducing non-bridging oxygen atoms to the system that facilitate vibration of molecules/atoms^106^. These can contribute to increased polarization under an applied electric field and consequently, the ɛʹ is increased. These structural changes are confirmed by the FTIR study, which is in agreement with the above-mentioned discussion. It has been reported that the dielectric constant of materials depends on the dielectric properties of each phase, the concentration, and the interfaces between different phases^107,109^. Therefore, the obtained results for glass ceramic samples BGC2 and BGC4 (Fig. 12b and Table 6) show the highest dielectric constant values due to the presence of BaTiO_3_ phase (15%) in these samples, which were confirmed by XRD (Fig. 1b).

Also, in Fig. 12b, there are observed peaks at about 100 Hz for samples BGC2 and BGC4. These peaks may be due to the presence of crystalline phases and the formation of resonance frequency between the applied field and the interphase boundaries in these samples^110,111^. Finally, the glass-ceramic exhibits a higher dielectric constant than the parent glass, suggesting the presence of interfacial polarization at the grain boundaries^112^ and the ɛʹ increases by the substitution of TiO_2_ at the expense of BaO for all the investigated glasses and glass ceramic samples.

**Fig. 13:**
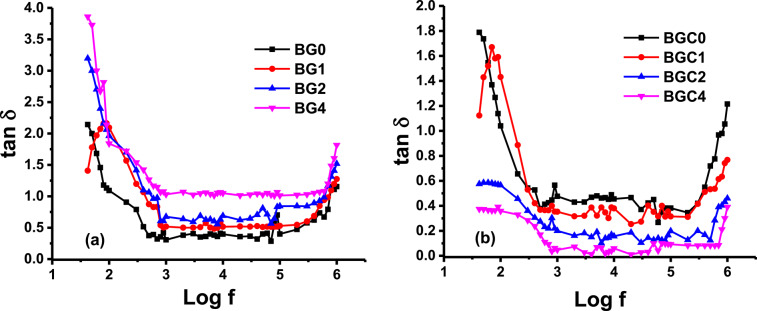
Variation of dielectric loss (tan δ) as a function of frequency for all samples (**a**) glasses and (**b**) glass ceramics at room temperature.

In Fig. 13, the dependence of dielectric loss tangent (tan δ) as a function of frequency for all the investigated glasses and glass ceramics is shown. From the figure, it is observed that the dielectric loss of the studied glass system increases with increasing TiO_2_, while the dielectric loss of the studied glass ceramics decreases with doping content. It has been reported that the higher the mobility of the conducting species, the higher the dielectric loss component^113,114^, so the replacement of BaO by TiO_2_ in the glasses leads to a more open and less strongly bonded network that tends to cause an increase in the dielectric losses^115^. But the more rigid structure caused by doping in the glass ceramics reveals the decrease in dielectric losses. In addition, the tan δ remains approximately stable in the frequency range of 1–500 kHz. This behavior is beneficial for ceramics used in electrical energy storage^116,117^.

**Table 5:** The AC activation energy (E_a(ac)_) at different frequencies for BG0 and BGC0 samples.

Samples Code		E_a(ac)_, (eV)					
		1 kHz	10 kHz	100 kHz	500 kHz	800 kHz	1 MHz
Glass	BG0	0.693	0.538	0.397	0.333	0.326	0.317
Glass ceramic	BGC0	0.446	0.374	0.300	0.283	0.220	0.172

**Table 6:** The DC activation energy (E_a(dc)_), the DC conductivity (σ_dc_) at room temperature (RT) and the dielectric constant (ɛʹ) at 1 khz and at RT for all samples.

Samples Code	Glasses				Glass ceramics			
	BG0	BG1	BG2	BG4	BGC0	BGC1	BGC2	BGC4
E_a(dc)_(eV)	0.728	0.12	0.065	0.053	0.462	0.107	0.035	0.037
σ_dc_, (S/cm)	9.82 × 10− 9	2.60 × 10^− 7^	8.62 × 10^− 7^	2.86 × 10^− 6^	2.04 × 10^− 10^	7.10 × 10^− 8^	6.05 × 10^− 7^	6.64 × 10^− 7^
ɛʹ, 1 kHz	42.17	204.31	304.31	393.28	94.95	308.55	454.58	477.21

### Biological activities study

#### Antimicrobial activity

**Fig. 14:**
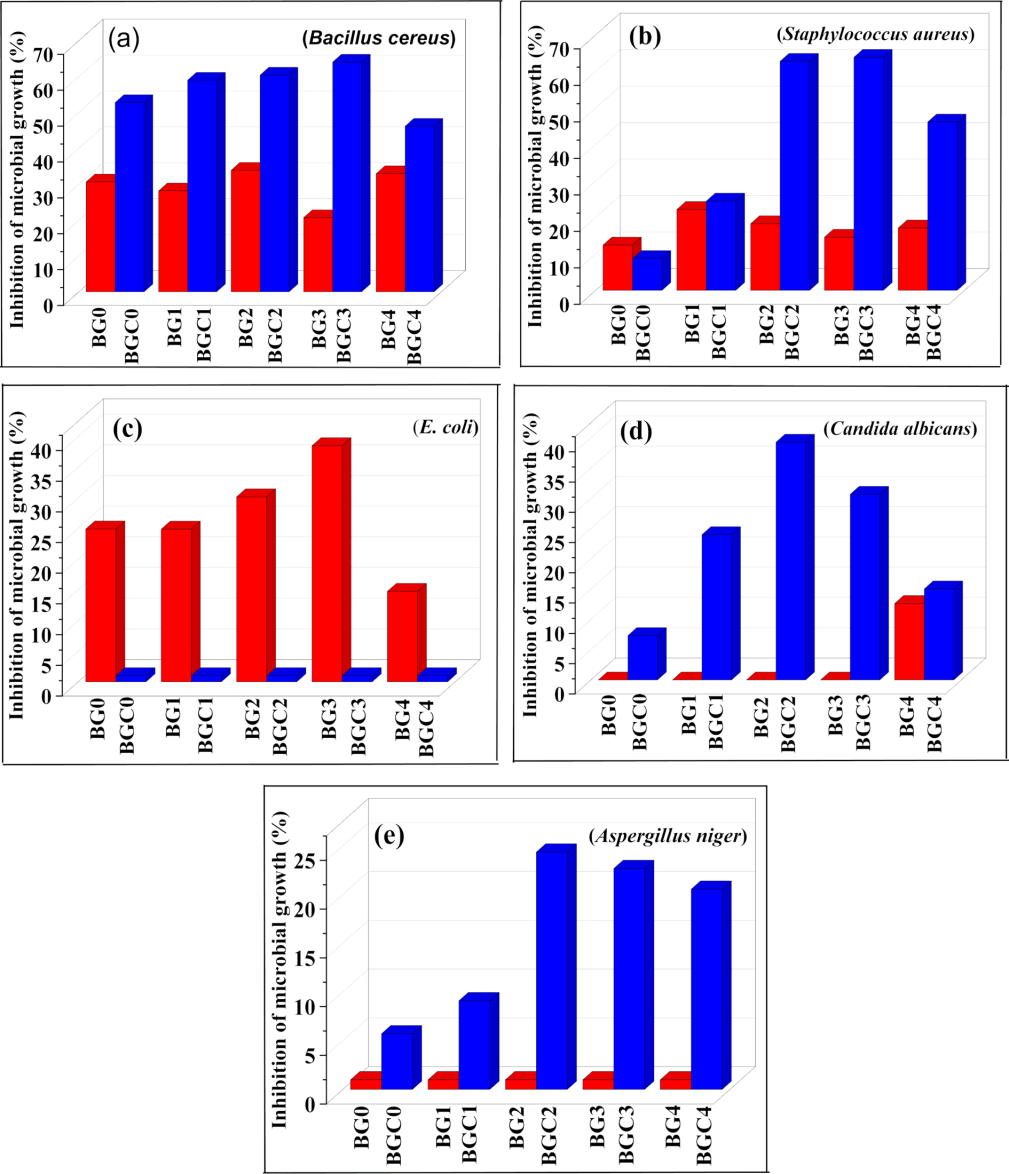
Antimicrobial activity of glass and glass ceramic samples (MIC).

In general, the results in Table 7; Fig. 14 showed that glass ceramic had more antimicrobial activity than glass samples and this result is in accordance with those reported by Helmy et al.^118^ and Ouis et al.^119^. It was noticed that, as the concentration of TiO_2_ content increased, the antimicrobial activity increased until definite concentration, after that, the activity decreased or still constant. Also, the antifungal activity against *Candida albicans* and *Aspergillus niger* and antibacterial activity against Bacillus cereus and Staphylococcus aureus of glass ceramic is preferable than glass samples, although the antimicrobial activity of glass against gram-negative bacteria (*E. coli*) was more than that of glass ceramic which had no activity against it, and this may be due to the hard structure of cell membrane of it. It was reported that boron components have antimicrobial activity against bacteria^120,121^ and fungi^122^. The antibacterial activity of boron may originate from its ability to rupture bacterial cell membranes through increasing release of free radicals (such as hydroxyl radicals ·OH) upon its reaction with H_2_O^123^. Also, the antimicrobial effect may be related to an increase in pH in culture medium (alkaline medium) arising from alkaline ions release (such as sodium) and hence elevation of osmotic pressure attributable to glass dissolution^124–126^. This increase in pH changes the integrity of the cytoplasmic membrane, promoting protein denaturation. The adjustment of intracellular pH might affect cellular functions, including the activity of enzymes essential for cellular metabolism^127^. Concerning the small portion UV of the indoor natural light photocatalytic effect, the TiO_2_ in borate glass generates reactive oxygen species (ROSs) such as hydroxyl radical (·OH), hydrogen peroxide (H_2_O_2_), superoxide radical anion (O_2_^−^) which cause oxidative damages to living organisms through lipid peroxidation of the cell membrane leading to the leach of proteins, minerals, and genetic materials^128^. Incorporation of B atoms into the TiO_2_ lattice (B-O-Ti as shown in FTIR) results in a charge imbalance and lattice distortion so TiO_2_ generates surface oxygen vacancy, which also improves TiO_2_ antimicrobial activity^129,130^. From the obtained data of this study, it was observed the preferable antimicrobial activity of glass-ceramics than glass samples by increasing TiO_2_ content. Our suggested assumption for this behavior is attributed to the thermal activation of TiO_2_ at high temperatures. Nishikawa et al. verified experimentally the formation of oxygen vacancies in TiO_2_ followed by the formation of trapped electrons and active radical species at high temperatures^131^. These oxygen vacancies and active radical species cause oxidative stress that can damage proteins, DNA, and lipids, resulting in microbial cell death^129,132^. Finally, the enhanced antimicrobial activity is accounted for the synergistic effect of TiO_2_ and B_2_O_3_ and these obtained results recommend samples for medical applications.

**Table 7:** Antimicrobial activity of glass and glass ceramic samples (MIC).

Samples Code		Inhibition of microbial growth (%)				
		Microbes				
		*Bacillus cereus*	*Staphylococcus aureus*	*E. coli*	*Candida albicans*	*Aspergillus niger*
Glass	BG0	30.65 ± 0.12	12.45 ± 0.06	24.90 ± 0.46	-	-
	BG1	28.16 ± 0.33	22.18 ± 0.17	24.87 ± 0.69	-	-
	BG2	33.90 ± 0.52	18.29 ± 0.26	30.14 ± 0.27	-	-
	BG3	20.69 ± 0.58	14.59 ± 1.00	38.49 ± 0.84	-	-
	BG4	32.95 ± 0.16	17.12 ± 0.85	14.72 ± 0.65	12.60 ± 0.19	-
Glass ceramic	BGC0	52.76 ± 0.52	8.75 ± 0.65	-	7.35 ± 0.84	5.72 ± 0.44
	BGC1	59.00 ± 0.85	24.50 ± 0.84	-	24.00 ± 0.65	9.11 ± 0.19
	BGC2	60.43 ± 0.24	62.80 ± 0.24	-	39.30 ± 0.17	24.36 ± 1.05
	BGC3	64.03 ± 0.63	63.90 ± 0.49	-	30.67 ± 0.39	22.67 ± 1.22
	BGC4	56.83 ± 1.15	46.17 ± 0.64	-	15.02 ± 0.75	20.55 ± 0.99

#### Longer-term stability assessments

**Table 8:** Antimicrobial activity of glass and glass ceramic samples after storage on broth media.

Samples Code		Inhibition of microbial growth (%)				
		Microbes				
		*Bacillus cereus*	*Staphylococcus aureus*	*E. coli*	*Candida albicans*	*Aspergillus niger*
Glass	BG0	49.62 ± 0.12	5.98 ± 1.23	-	4.87 ± 0.55	3.25 ± 0.14
	BG1	55.21 ± 0.32	22.55 ± 0.26	-	20.63 ± 0.74	5.37 ± 0.85
	BG2	57.36 ± 0.57	60.70 ± 0.47	-	31.31 ± 0.96	20.63 ± 0.99
	BG3	60.15 ± 1.00	64.54 ± 0.87	-	20.35 ± 0.21	15.69 ± 0.41
	BG4	53.24 ± 1.06	45.12 ± 0.64	-	11.23 ± 0.32	12.00 ± 1.20
Glass ceramic	BGC0	48.56 ± 0.32	49.20 ± 0.74	17.21 ± 0.54	25.63 ± 1.32	14.20 ± 0.45
	BGC1	48.74 ± 0.21	50.35 ± 0.52	20.60 ± 0.84	37.52 ± 0.87	33.58 ± 0.95
	BGC2	50.00 ± 0.52	58.14 ± 0.14	28.63 ± 0.41	50.54 ± 1.11	45.32 ± 0.74
	BGC3	50.20 ± 0.41	58.63 ± 0.63	49.63 ± 0.16	42.51 ± 0.95	44.44 ± 0.63
	BGC4	55.21 ± 0.98	60.00 ± 0.99	22.41 ± 0.10	34.62 ± 0.12	18.32 ± 0.21

The results observed in Table 8 indicated that the antimicrobial activity of samples stored for approximately one year remained nearly unchanged compared to freshly prepared samples. The glass ceramic was more stable than the glass, even after storage. These results recommended high stability of both glass and glass ceramic samples after approximately one year of storage where some samples of glass ceramic retained 100% of their activity after storage. The durability of the antimicrobial effect indicates the stability of the prepared glass and glass ceramics and the glass matrix successfully preserved its functionality So they are recommended in medical, pharmaceutical, and industrial applications.

#### Cytotoxicity evaluation

**Table 9:** Values of cell Viability % with different concentrations of glass (BG0, BG2, and BG4) and glass ceramic (BGC0, BGC2, and BGC4) samples.

Sample conc. (µg/ml)	Cell Viability %					
	Glass			Glass ceramics		
	BG0	BG2	BG4	BGC0	BGC2	BGC4
1000	21.05±2.13	38.61±2.35	24.59±2.17	23.46±1.68	14.78±1.26	11.42±0.84
500	45.13±2.71	49.85±1.74	47.21±1.43	46.78±2.05	39.57±1.91	30.61±1.25
250	76.91±1.45	88.04±0.82	80.69±1.85	79.24±1.48	62.35±1.83	48.19±1.76
125	92.40±0.68	99.72±0.17	94.13±0.69	92.95±0.71	86.04±1.27	78.65±1.23
62.5	98.17±0.46	100	98.96±0.22	99.04±0.13	93.76±0.62	91.34±0.29
31.25	100	100	100	100	99.86±0.39	98.12±0.64
15.6	100	100	100	100	100	100
7.8	100	100	100	100	100	100
0	100	100	100	100	100	100

**Fig. 15:**
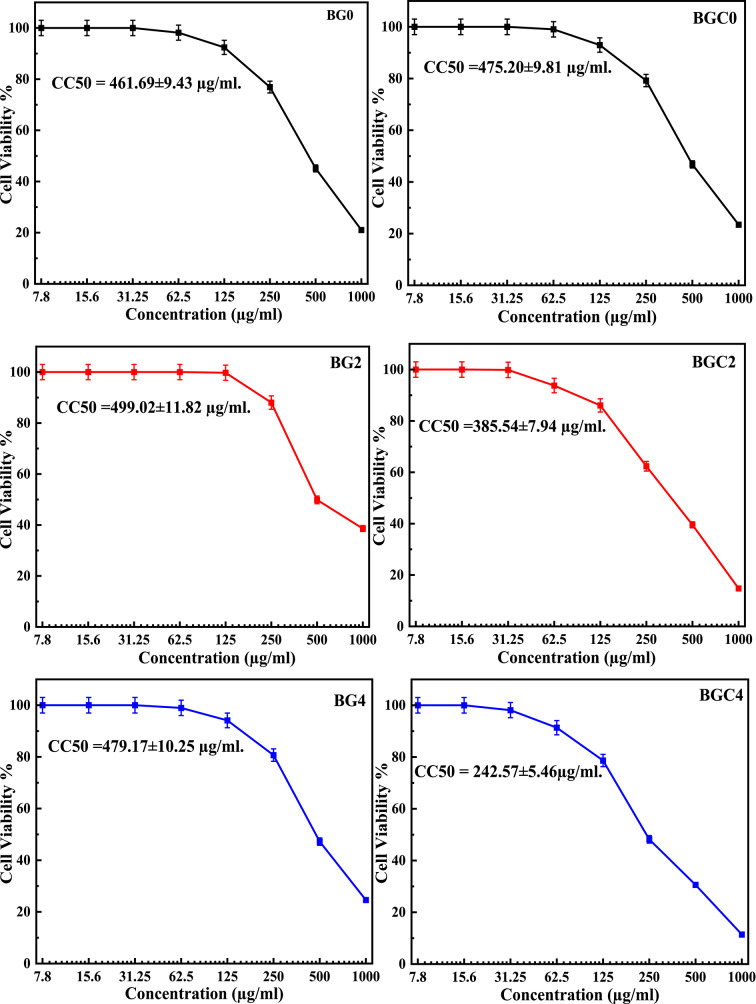
Effect of different concentrations of glass (BG0, BG2, and BG4) and glass ceramic (BGC0, BGC2, and BGC4) samples on Cell Viability % and CC_50_.

Cell viability % of selected glass (BG0, BG2, and BG4) and their corresponding glass ceramic (BGC0, BGC2, and BGC4) samples was estimated in the cell culture media for 24 h against Human normal melanocytes cell line (HFB 4 cells) as shown in Table 9. The relation between cell viability % against different concentrations of glass and glass ceramics (7.8–1000 µg/ml) is plotted to get the survival curve of the tested cell line after treatment with samples (Fig. 15). The cytotoxicity concentration CC_50_, the concentration required to cause toxic effects in 50% of intact cells, was also demonstrated in Fig. 15. As demonstrated, the cell viability was affected by both the concentrations of the samples and the content of TiO_2_. It is worth mentioning that all synthesized glass and glass ceramics exhibit approximately 100% cell viability at concentrations ranging from 7.8 to 31.25 µg/ml and (91.34–99.72%) cell viability at concentrations up to 125 µg/ml (except for BGC2 and BGC4). Unlike results reported in^133^, the glass samples have minimal cytotoxicity compared with their corresponding glass ceramics. The values of cell viability obtained in this study are good compared to the cell viability values reported in^133^. It can be confirmed that the prepared glass and glass ceramics samples were nontoxic in nature, because the cell viability is greater than 70% ^47^ so, these results suggest that the glass and glass-ceramic samples had lower to normal cytotoxicity at concentration up to 250 µg/ml (except the BGC2 and BGC4 samples that had lower cytotoxicity at concentration up to 125 µg/ml) and could be safely used as biocompatible materials.

## Conclusion

Briefly, a series of TiO_2_-modified borate glass compositions (40 B_2_O_3_-20 Al_2_O_3_-10 NaF-(30-X) BaO-X TiO_2_) where, (0 ≤ X ≤ 2.5) mol% were prepared by melt quench method to study the effect of gradual addition of TiO_2_ at expense of BaO on structural, electrical and antimicrobial properties. Also, the corresponding glass-ceramics were synthesized by heat treatment at a nucleation temperature of 480℃ for 4 h and a crystal growth temperature of 750℃ with a rate of 5 °C/min for 6 h. FTIR study showed the appearance of titanium ions in the network of the glass, mainly as TiO_4_ (network former) at lower concentrations of TiO_2_ and TiO_6_ (network modifier) with a gradual increase in TiO_2_ content. For glass-ceramics, FTIR showed sharp and split peaks due to the fine crystalline derivatives, which were also confirmed by XRD and SEM measurements. A slight decrease in density from 3.494 g/cm^3^ to 3.273 g/cm^3^ and an expansion in the molar volume from 28.173cm^3^/mol to 29.627cm^3^/mol were caused as a result of the substitution of BaO by TiO_2_ in the glass network. The electrical conductivity of the glass and glass ceramics is thermally activated and follows Arrhenius’ behaviour. The calculated activation energy lies in the range of 0.053–0.728 (eV) and 0.037 − 0.462 (eV) for glass and glass ceramic samples, respectively. The DC conductivity values of the studied samples indicated that the incorporation of TiO_2_ results in higher conductivity values than those of the free samples. The prepared glasses doped with TiO_2_ and its glass ceramics exhibited a semiconducting nature and the magnitude of the ơ_dc_ values indicated that the electrical conductivity of such samples lies in the range of semiconductors. The glass-ceramic exhibits higher dielectric constants than the parent glass, and it increases by doping with TiO_2_. Due to values of electrical conductivity and ɛʹ, the present samples can be considered as good candidates for energy storage and are one of the semiconductor categories that can be used in electronic devices. The prepared doped- TiO_2_ borate glass and glass ceramics were found to have biological activity against various species of microbes, but glass ceramic is more preferable than the parent one. The results recommended high stability of both glass and glass ceramic samples after approximately one year of storage. The prepared glass and glass ceramics samples were nontoxic in nature and the glass samples have minimal cytotoxicity compared with their corresponding glass ceramics. Finally, the unique fabrication of promising multifunctional materials of TiO_2_-doped borate glasses and glass-ceramics can meet the demands of both biological and electrical applications.

## Data Availability

Availability of Data& MaterialsThe data of this work is available for any person after publication. For any questions about the data from this study contact the corresponding author (Gehad Y. Abo El-Reesh).
